# The Relationship Between Smoker Identity and Smoking Cessation Among Young Smokers: The Role of Smoking Rationalization Beliefs and Cultural Value of Guanxi

**DOI:** 10.3389/fpsyt.2022.812982

**Published:** 2022-04-26

**Authors:** Haide Chen, Yumeng Fan, Xinwei Li, Lingfeng Gao, Weijian Li

**Affiliations:** College of Education and Human Development, Zhejiang Normal University, Jinhua, China

**Keywords:** smoker identity, smoking cessation behavior, smoking rationalization beliefs, intention to quit smoking, cultural value of guanxi, young smokers

## Abstract

Although the relationship between smoker identity and smoking cessation behavior has been confirmed, the role of smoking-related beliefs and cultural values in this relationship for young smokers is little known. The present study aimed to examine whether the relationship between smoker identity and smoking cessation behavior would be mediated by smoking rationalization beliefs and/or intention to quit smoking and whether the effect of smoker identity on smoking cessation behavior was moderated by cultural value of guanxi. A total of 708 young smokers participated in the study and completed questionnaires that measured smoker identity, smoking rationalization beliefs, intention to quit smoking, smoking cessation behavior and cultural value of guanxi. The results showed: (1) the relationship between smoker identity and smoking cessation behavior was negative and significant. (2) The mediating effect of intention to quit smoking and the serial mediating effect of “smoking rationalization beliefs → intention to quit smoking” on the relationship between smoker identity and smoking cessation behavior was significant. (3) Both the serial mediating effect of “smoking rationalization beliefs → intention to quit smoking” and the direct effect of smoker identity on smoking cessation behavior were moderated by cultural value of guanxi. The current findings increased understanding of psychosocial mechanisms underlying the hindering effect of smoker identity on smoking cessation and suggested the role of smoking rationalization beliefs and cultural value of guanxi should be considered in smoking cessation interventions for young smokers.

## Introduction

Tobacco use is one of the preventable and life-threatening public health problems worldwide. Quitting smoking before middle age would reduce the risks of smoking-induced morbidity and mortality (1). However, young smokers often consider their nicotine dependence lower than older smokers' nicotine dependence, and therefore their intention to quit smoking is generally lower than that among older smokers (2, 3). It is well-known that intention to quit smoking is the antecedent of taking specific actions to quit smoking [e.g., (4)]. A study showed that smoking cessation behavior was significantly predicted by intention to quit smoking (5). In recent years, smoker identity has received more and more attention to gain a deeper understanding of the process of smoking cessation. However, it is still little known about the underlying psychosocial mechanism of the effect of smoker identity on intention to quit smoking and smoking cessation behavior, especially among young smokers.

### Smoker Identity and Smoking Cessation

PRIME theory defines identity as the mental representations of ourselves and the thoughts and feelings we attach to these (6). Smoker identity refers to the degree to which smokers internalize smoking behavior as a defining aspect of themselves (7), may reduce the intention to quit smoking and the frequency of smoking cessation behavior. In terms of the effect of identity on behavioral intention, it is found that the positive feelings of being a smoker can reduce the intention to quit smoking (8). The Theory of Planned Behavior (TPB) is one of the classical models to explain the mechanism of health behavior, which has been widely applied to predict smoking cessation in previous studies (9–11). The TPB suggests that three factors (i.e., attitudes, subjective norms, perceived behavioral control) influence behavioral intention, which in turn influence behavioral performance (4). In addition, an extended version of the theory of planned behavior has shown that the predictive power of identity on behavioral intention was higher than that of the factors involved in the Theory of Planned Behavior (12). In terms of the effects of identity on behavior, it is also found that individuals who identify as smokers are more likely to increase the frequency of smoking (13) and to reduce quit attempts (7).

### The Role of Smoking Rationalization Beliefs

Numerous studies have shown that cognitive factors influence smoking cessation. We should take into account the role of smoking-related beliefs when explain the effect of identity on smoking cessation. As one of the common beliefs existing among smokers, smoking rationalization beliefs, which refer to beliefs that enhance the functional features or minimize the negative features of smoking (14), were indicated as an obvious barrier to smoking cessation (15). According to Cognitive Dissonance Theory (16), psychological discomfort may arise when individuals' behavior and cognition are inconsistent. To avoid and reduce the discomfort caused by such cognitive dissonance, either belief or behavior would be chosen to change. As smoking beliefs are relatively easy to change compared to smoking cessation behavior, smokers with dissonance prefer to choose smoking beliefs change (i.e., replacing smoking hazard beliefs with smoking rationalization beliefs). The emerging smoking rationalization beliefs would reduce smokers' motivation to quit smoking. Previous studies showed that smoking rationalization beliefs caused smokers to deny the risks of smoking and thus reduced their intention to quit smoking (17) and prevented the implementation of smoking cessation behavior (18).

Identity may enhance positive cognition of self-related behavior, as it motivates individuals to form certain rationalization beliefs which consistent with their identity (19). For smokers who identify themselves as smokers, smoking is a prominent label of their self-identity (7). Moreover, rationalization of smoking behavior is one of the accessible and effective strategies to positively define smoking behavior among smokers (18). Given these, smoking rationalization beliefs might be associated with developed smoker identity.

### The Moderating Role of Cultural Value of Guanxi

The influence of cultural value is necessary to be considered in the study of individual behavior (20–22). For example, an international tobacco control survey has shown that the difference in quit intentions among smokers was, in part, explained by the cultural value (21). According to Schwartz (23), a collectivist society is a communal society characterized by mutual obligations and expectations based on ascribed statuses. Collectivist culture emphasizes interdependence, social embeddedness, and obligations and loyalty to in-group (24, 25). Some of the East Asian societies (e.g., Korea, Japan, China) tend to be interdependent, as indicated by a relatively greater focus on harmonious connections (26). Guanxi is an indigenous Chinese concept, which generally refers to relationships or social connections based on mutual interests and benefits (27). Individuals holding cultural value of guanxi are likely to perform behaviors that facilitate the relationship with others (28). The tendency of individuals to engage in guanxi-building activities (e.g., attend a banquet) reflects their cultural value of guanxi (27).

Similarly, smokers holding cultural value of guanxi may attach great importance to harmonious relationships, especially with in-group members. For smokers, smoking is considered as a process of social activities. Sharing a cigarette or a light and smoking with others may contribute to guanxi establishment and social interaction with other smokers (29). Given this, smoking cessation may be influenced by cultural value of guanxi. Rejecting cigarettes from others may be contrary to smokers' self-identity as smokers, which might lead to the breakdown of the connection with members of smoker groups (30). Owing to the negative smoking refusal outcome expectations (e.g., “the group would no longer be my friends.”), smokers who hold a high degree of cultural value of guanxi are less likely to reject the cigarettes offered by other smokers in a social context (31). According to the above evidence, the present study speculates that cultural value of guanxi may magnify the hindering effect of smoker identity on smoking cessation.

### The Present Study

According to the literature base, we hypothesized that smoker identity was negatively associated with smoking cessation behavior (H_1_), the relationship between smoker identity and smoking cessation behavior was mediated by smoking rationalization beliefs and intention to quit smoking (H_2_), and the effect of smoker identity on smoking cessation behavior was moderated by cultural value of guanxi (H_3_).

## Methods

### Participants

Current smokers defined as those who had smoked at least 100 cigarettes in their life and had smoked at least one cigarette in the past 30 days were recruited to participate in the survey voluntarily. Participants were recruited by convenience sampling. Specifically, participants were recruited via advertisements posted on social networking sites in the local university and community. Inclusion criteria were: (1) being 18–35 years of age, (2) have smoked at least 100 cigarettes in life (32) and have smoked at least one cigarette in the past 30 days (33), and (3) did not have a history of other drug use. After voluntarily signing up for the survey, participants underwent an interview to confirm whether they met the inclusion criteria. A total of 708 young smokers validly completed the questionnaire and their data were selected to analyze in the present study. The average age of becoming a daily smoker was 19.42 (*SD* = 4.04) and the number of cigarettes they smoked per day was 10.02 (*SD* = 7.60). Demographic Characteristics of participants were shown in Table 1.

**Table 1:** Demographic characteristics.

**Characteristic**	***N* = 708**	**Unweighted %**
**Age**		
18–25	449	63.42
26–30	156	22.03
31–35	103	14.55
**Gender**		
Male	642	90.68
Female	66	9.32
**Marital status**		
Unmarried	543	76.69
Married	165	23.31
**Socioeconomic status**		
3–9	367	51.84
10–15	320	45.20
16–21	21	2.97

*The socioeconomic status index is the sum of scores for three variables (i.e., income, education and position rank). All three variables are scored on 7-point Likert scales. A higher socioeconomic status index corresponds to higher socioeconomic status*.

### Measures

#### Smoker Identity

Smoker identity was measured using an adaptation of the Smoker Identity Scale developed from previous studies (10, 34). The scale comprised 4 items (e.g., “*I am a good example of a person who smokes*.”). Participants responded on a 7-point Likert scale (1= completely disagree, 7= completely agree). The higher the scores, the higher the degree to which individuals identified themselves as smokers. In the present study, Cronbach's α for this scale was 0.81.

#### Smoking Rationalization Beliefs

Smoking rationalization beliefs were measured using the Smoking Rationalization Beliefs Scale (35). The scale comprised 26 items (e.g., “*Smoking is good for inspiration and active thinking*.”) and was divided into 6 dimensions including smoking functional beliefs, risk generalization beliefs, social acceptability beliefs, safe smoking beliefs, self-exempting beliefs and quitting is harmful beliefs. Participants responded on a 5-point Likert scale (1 = completely disagree, 5 = completely agree). Higher scores indicated a higher level of smoking rationalization beliefs. In the present study, Cronbach's α for this scale was 0.89.

#### Intention to Quit Smoking

Intention to quit smoking was measured using two items (36, 37). One of the items is “*In the last month, how strong is your desire to quit smoking*”, which is scored on a 5-point Likert scale that ranged from 1 (not at all) to 5 (very much). The other is “*When do you intend to quit smoking*,” which is scored on a 5-point Likert scale that ranged from 1 to 5 (1 = have never thought about quitting, 2 = within 2 years, 3 = within 6 months, 4 = within 2 months, 5 = within 1 month). The higher the average score of the two items, the stronger the intention to quit smoking.

#### Smoking Cessation Behavior

Smoking cessation behavior was measured using a single item (i.e., “*How many days have you quit smoking for the last month?*”) which was developed based on a review of the literature on quitting smoking (11, 38, 39). The more days of quitting smoking they reported, the better they performed in smoking cessation.

#### Cultural Value of Guanxi

Cultural value of guanxi was measured using the subscales from Qian et al. (40), which comprised 13 items (e.g., “*I believe that developing guanxi is necessary in one's daily life*.”). Participants responded on a 7-point Likert scale (1 = completely disagree, 7 = completely agree). Higher scores indicated that individuals held a higher degree of cultural value of guanxi. In the present study, Cronbach's α for this scale was 0.87.

#### Nicotine Dependence

Nicotine dependence was measured using the Fagerström Test for Nicotine Dependence Scale [FTND; (41)] which comprised 6 items (e.g., “*Whether it is difficult to refrain from smoking in places where it is forbidden?*”). Higher scores indicated that smokers had a higher degree of nicotine dependence.

### Procedure

The study protocol was approved by the Institutional Review Board of a local university in China. Informed consent was obtained from all of the participants. Then, participants were guided on how to complete the questionnaires and completed the online survey anonymously within ~15 min.

### Statistical Analyses

Firstly, Harman's one-way test was conducted to examine the common method biases and the result showed that there were 8 factors with eigenvalues over 1. The amount of variation explained by the first factor was 18.51%, which indicated that common method biases were not serious in the present study. Then, the serial multiple mediation analysis and the moderated serial multiple mediation analysis were performed, respectively, by Model 6 and 92 of PROCESS macro (42) in SPSS. The standard error and 95% confidence interval of the parameter estimation were obtained by bootstrapping method (repeated sampling 5,000 times). In addition, simple slope analyses were performed.

## Results

### Correlation Analyses of the Major Variables

The correlations of the major variables were analyzed using partial correlation analysis controlling for factors including gender, age, marital status, socioeconomic status and nicotine dependence. These factors are robust and common predictors within the domain of tobacco research [e.g., (43, 44)]. As shown in Table 2, smoker identity was positively associated with smoking rationalization beliefs but negatively associated with intention to quit smoking and smoking cessation behavior. Smoking rationalization beliefs were negatively associated with intention to quit smoking. Intention to quit smoking was positively associated with smoking cessation behavior.

**Table 2:** Means, standard deviations and partial correlations among variables.

	***M***	***SD***	**1**	**2**	**3**	**4**	**5**
1 Smoker identity	4.66	1.28	-				
2 Smoking rationalization beliefs	2.78	0.58	0.14^***^	-			
3 Intention to quit smoking	2.64	1.26	−0.18^***^	−0.14^***^	-		
4 Smoking cessation behavior	4.04	7.16	−0.27^***^	−0.04	0.35^***^	-	
5 Cultural value of guanxi	4.91	0.93	0.24^***^	0.12^**^	−0.08^*^	−0.04	-

* *p < 0.05*,

** *p < 0.01*,

*** *p < 0.001*.

### The Serial Multiple Mediation Model

The serial multiple mediation model was analyzed using Model 6 of PROCESS macro (42) after controlling for some factors (e.g., gender). The results were as follows. The total effect of smoker identity on smoking cessation behavior was −1.59 (95% CI = −2.02, −1.17), the direct effect was −1.28 (95% CI = −1.70, −0.87), the indirect effect was −0.31 (95% CI = −0.49, −0.15) which comprised 19.5% of the total effect. In the indirect effect, the coefficient of the pathway for “smoker identity → smoking rationalization beliefs → smoking cessation behavior” was 0.03 (95% CI = −0.03, 0.09), the coefficient of the pathway for “smoker identity → intention to quit smoking → smoking cessation behavior” was −0.30 (95% CI = −0.48, −0.15), and the coefficient of the sequential pathway for “smoker identity → smoking rationalization beliefs → intention to quit smoking → smoking cessation behavior” was −0.03 (95% CI = −0.06, −0.01). These results indicated that the pathway for “smoker identity → intention to quit smoking → smoking cessation behavior” was significant, indicating that intention to quit smoking mediated the relationship between smoker identity and smoking cessation behavior. Similarly, the sequential pathway for “smoker identity → smoking rationalization beliefs → intention to quit smoking → smoking cessation behavior” was significant, implying that higher degree of smoker identity was serially associated with higher level of smoking rationalization beliefs, lower intention to quit smoking, as well as less smoking cessation behavior.

### The Moderated Serial Mediation Model

**Figure 1:**
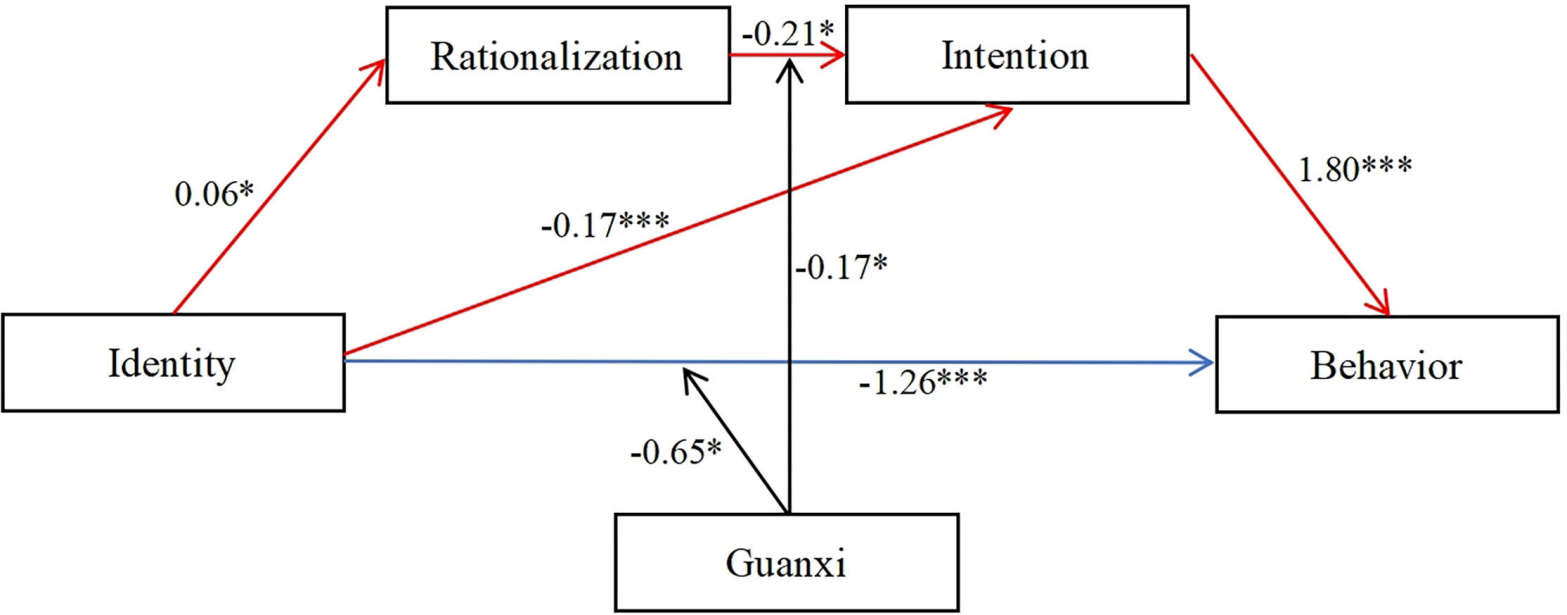
The moderated serial multiple mediation model. *p < 0.05, **p < 0.01, ***p < 0.001.

A moderated serial mediation analysis was further conducted using Model 92 (see Figure 1). As shown in Table 3, intention to quit smoking was significantly and negatively predicted by the interaction terms of smoking rationalization beliefs and cultural value of guanxi. The conditional indirect effect of the pathway for “smoker identity → smoking rationalization beliefs → intention to quit smoking → smoking cessation behavior” was further examined. As shown in Table 4, the indirect effect was significant (*B* = −0.04, 95% CI = −0.11, −0.00) for smokers with high degree of cultural value of guanxi (*M* + *1SD*) and the indirect effect was not significant (*B* = −0.00, 95% CI = −0.03, 0.02) for smokers with low degree of cultural value of guanxi (*M*–*1SD*). Similarly as shown in Table 3, smoking cessation behavior was significantly and negatively predicted by the interaction items of smoker identity and cultural value of guanxi. The conditional direct effect of the pathway for “smoker identity → smoking cessation behavior” was further examined. As shown in Table 4, the direct effect was greater among smokers with high degree of cultural value of guanxi (*B* = −1.86, 95% CI = −2.39, −1.34) than those with low degree of cultural value of guanxi (*B* = −0.66, 95% CI = −1.24, −0.07).

**Table 3:** The summary of the moderated serial multiple mediation model.

	**M**_**1**_			**M**_**2**_			**Y**		
	***B***	***SE***	**95%CI**	***B***	***SE***	**95%CI**	***B***	***SE***	**95%CI**
X	0.06**	0.02	0.02, 0.10	−0.17^***^	0.04	−0.25, −0.09	−1.26^***^	0.22	−1.68, −0.84
M_1_	–	–	–	−0.21*	0.08	−0.37, −0.05	0.30	0.43	−0.54, 1.14
M_2_	–	–	–	–	–	–	1.80^***^	0.20	1.41, 2.19
W	0.06*	0.03	0.01, 0.11	−0.03	0.05	−0.13, 0.08	0.24	0.28	−0.30, 0.79
X*W	0.01	0.02	−0.03, 0.04	0.03	0.04	−0.04, 0.11	−0.65*	0.20	−1.04, −0.26
M${}_{1}^{*}$W	–	–	–	−0.17*	0.09	−0.34, −0.01	0.33	0.44	−0.54, 1.20
M${}_{2}^{*}$W	–	–	–	–	–	–	0.04	0.22	−0.40, 0.48
U_1_	0.01	0.01	−0.01, 0.02	−0.04**	0.02	−0.07, −0.01	0.05	0.08	−0.11, 0.21
U_2_	0.11	0.08	−0.04, 0.26	−0.14	0.16	−0.46, 0.18	1.23	0.85	−0.44, 2.91
U_3_	0.02	0.06	−0.11, 0.15	−0.11	0.14	−0.38, 0.16	0.39	0.72	−1.01, 1.80
U_4_	0.01	0.01	−0.01, 0.02	0.01	0.02	−0.03, 0.04	−0.06	0.09	−0.23, 0.11
U_5_	0.02	0.01	−0.00, 0.04	−0.03	0.02	−0.07, 0.02	−0.42^***^	0.12	−0.65, −0.19
C	−0.43**	0.18	−0.78, −0.08	1.23**	0.38	0.47, 1.98	3.20	2.02	−0.76, 7.16
	*R^2^ =* 0.06, *F* (8, 699) *=* 5.08			*R^2^ =* 0.10, *F* (10, 697) = 7.69			*R^2^ =* 0.25, *F* (12, 695) *=* 19.49		

* *p < 0.05*,

** *p < 0.01*,

*** *p < 0.001*.

*X, Smoker identity; M_1_, Smoking rationalization beliefs; M_2_, Intention to quit smoking; W, Cultural value of guanxi; U_1_, Age; U_2_, Gender; U_3_, Marital status; U_4_, Socioeconomic status; U_5_, Nicotine dependence; C, Constant*.

**Table 4:** The pathways of the moderated serial multiple mediation model.

	**Indirect effect**			**Indirect effect**			**Direct effect**		
	**(X → M_2_→Y)**			**(X → M_1_→M_2_→Y)**			**(X → Y)**		
	***B***	***SE***	**95%CI**	***B***	***SE***	**95%CI**	***B***	***SE***	**95%CI**
W:*M-1SD*	−0.35	0.12	−0.61, −0.15	−0.00	0.01	−0.03, 0.02	−0.66	0.30	−1.24, −0.07
W:*M+1SD*	−0.25	0.11	−0.48, −0.05	−0.04	0.03	−0.11, −0.00	−1.86	0.27	−2.39, −1.34

**Figure 2:**
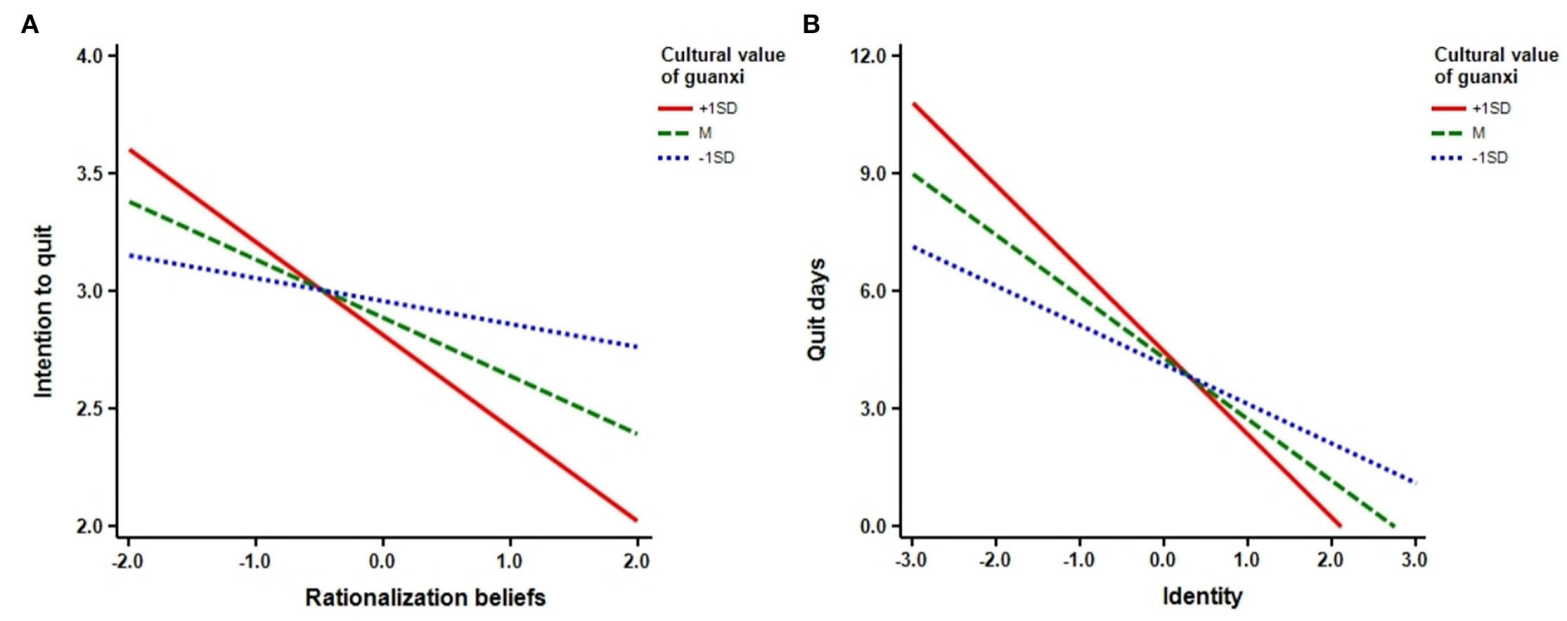
**(A,B)** Simple slope analyses.

In addition, simple slope analyses were performed. The interaction graphs were shown in Figure 2. Here, the left interaction graph reveals a negative relationship between smoking rationalization beliefs and intention to quit smoking for those who are high (1 SD above mean) on cultural value of guanxi (*B* = −0.40, *p* < 0.05), whereas there is no relationship between smoking rationalization beliefs and intention to quit smoking for those who are low (1 SD below mean) on cultural value of guanxi (*B* = −0.10, *p* > 0.05). The right interaction graph reveals a negative relationship between smoker identity and smoking cessation behavior for those who are high (1 SD above mean) on cultural value of guanxi (*B* = −2.12, *p* < 0.05) and those who are low (1 SD below mean) on cultural value of guanxi (*B* = −1.00, *p* < 0.05).

## Discussion

The present study found that smoking cessation behavior was negatively predicted by smoker identity among young smokers, which was consistent with the findings of previous studies [e.g., (7)]. The present study further found that the relationship between smoker identity and smoking cessation behavior was mediated by “smoking rationalization beliefs → intention to quit smoking.” Previous studies have also found multivariate correlations between these variables were significant (17, 19, 45). The mediating role of smoking rationalization beliefs in the relationship between smoker identity and smoking cessation might be explained as follows. Firstly, rationalization is conducive to alleviate the discomfort caused by unconscious personality components. According to Freud's hypothesis, rationalization is one of the psychological defense mechanisms that could relieve the anxiety brought about by the conflict between id and superego (46). For smokers, id follows the hedonic principle and satisfies the desire to smoke at all costs, however, superego follows the moral principle and resists the urge to smoke. Conflicts arising from the inconsistency between id and superego would be mitigated by rationalization. Secondly, according to the Cognitive Dissonance Theory (16), cognitive dissonance can be reduced by increasing the coordinated cognitive components. When a conflict occurs between smoking hazard information and smoker identity, smokers may react defensively to persuasive smoking hazard information (47). Specifically, alternative smoking rationalization beliefs that are compatible with smoker identity may emerge to relieve smokers' tension. In conclusion, the smoking rationalization beliefs may not only make smokers recognize the function of smoking (e.g., stress management), but also reduce their susceptibility to the hazard of smoking. Thus, these beliefs may decrease smokers' intention to quit smoking and reduce smoking cessation behavior.

The present study also found the moderating role of cultural value of guanxi in the relationship between smoking rationalization beliefs and intention to quit smoking was negative. Specifically, the predictive effect of smoking rationalization beliefs on intention to quit smoking was significant among smokers with a high degree of cultural value of guanxi rather than among smokers with a low degree of cultural value of guanxi. In other words, smoking rationalization beliefs, such as social acceptability beliefs and smoking functional beliefs, were activated by cultural value of guanxi, which then weakened smokers' intention to quit smoking. This result might be explained by two reasons. Firstly, smokers with a higher degree of cultural value of guanxi may be influenced by smoking peers (48). The prevalence of smoking behavior among peer groups may lead smokers holding cultural value of guanxi to believe that smoking is acceptable behavior for others. Then, social acceptability beliefs are activated and influence smokers' intention to quit smoking. Secondly, smokers with a higher degree of cultural value of guanxi may implement more guanxi-oriented behaviors (28). In other words, individuals who attach great importance to relationships tend to take practical actions to establish and promote their relationships. Smokers usually believe that offering cigarettes actively, accepting cigarettes and smoking together would make the relationship harmonious (29). They regard smoking as a means to develop the relationship and believe that smoking can promote social interaction, then their smoking functional beliefs are activated, further, influence their intention to quit smoking.

In addition, the present study found the moderating role of cultural value of guanxi in the relationship between smoker identity and smoking cessation behavior is significant and negative. The predictive effect of smoker identity on smoking cessation behavior is greater among smokers with a higher degree of cultural value of guanxi compared to those with a lower degree of cultural value of guanxi. This result might be explained by two reasons. Firstly, as for smokers with a high degree of cultural value of guanxi, they usually attach great importance to the established relationship with the smoking groups to obtain the sense of belonging of the smoking groups. However, smoking cessation behavior which is usually considered as a potential indicator of exclusion from smoking groups (30) would cause the existing relationship with other smokers to break up. Thus, with regard to smokers who identify themselves as smokers, the holding of cultural value of guanxi may increase the likelihood that smokers may take more smoking to protect their smoker identity. Secondly, smokers with a higher degree of cultural value of guanxi tend to implement more guanxi-oriented behaviors (28). Smokers usually take the initiative in passing cigarettes and smoking together to develop the guanxi with other smokers (29). Therefore, among smokers with a high degree of cultural value of guanxi, those who identify as a smoker may use smoking more to develop their guanxi, and they may be less likely to quit smoking.

In general, this study further explored the underlying mechanism of smoker identity on smoking cessation behavior by establishing a moderated serial multiple mediation model, which has certain theoretical value and practical significance. In theory, from the perspective of smoking rationalization beliefs, this study is conducive to understand “how” smoker identity affects smoking cessation behavior; from the perspective of Theory of Planned Behavior (4), it adds the explanation of health behaviors. The present study also focused on “when” the effect was greater and examined the effect of cultural value of guanxi, which could be contribute to developing a theory of smoking cessation behavior that fits the characteristic of smokers who hold high degree of cultural value of guanxi. In practice, the results of this study have some implications for the intervention of smoking behavior among young smokers. First of all, smokers need to change their identity as smokers, which may help to reduce smoking behavior. Secondly, it is necessary to reduce the spread of smoking misinformation (e.g., “*Smoking high-quality cigarettes is not harmful to health*.”). Finally, attention should also be paid to the specific effect of cultural value of guanxi on smokers. Cultural value of guanxi not only activates smoking rationalization beliefs but also expands the effect of smoker identity as an obstacle to smoking cessation. Therefore, it is necessary to reduce smokers' guanxi-oriented smoking behavior and avoid smoking cessation difficulties caused by using smoking as a means of social interaction.

There are still a few limitations in this study. Firstly, this was a cross-sectional survey study. In the future, a longitudinal study design is needed to follow the smoking cessation process of smokers. Specifically, smokers who are quitting smoking completes the baseline data for the study variables (i.e., smoker identity, smoking rationalization beliefs, intention to quit smoking, smoking cessation behavior, cultural value of guanxi) and self-reports data on these variables during the subsequent smoking cessation process (e.g., 2-year follow-up). Secondly, there are internal differences in smoking behavior among smokers. In future studies, more stringent inclusion criteria should be adopted to distinguish different types of smokers (e.g., social smoker). Thirdly, the recruitment source has been used as a covariate in previous studies [e.g., (49)]. In our study, the participants were recruited by social network and surveyed online, which was not random sampling method. So, there are some sampling biases. Future studies are expected to adopt random sampling method to avoid these biases. Finally, although this study focused on the effect of smoking rationalization beliefs and cultural value of guanxi, other cognitive and social environmental variables are still significant and may play roles in the relationship between smoker identity and smoking cessation.

## Conclusions

The findings indicate that smoking rationalization beliefs and cultural value of guanxi may play an important role in the relationship between smoker identity and smoking cessation, and then provide more implications for the cognitive intervention program such as cognitive behavioral treatment (50) of smoking behaviors and smoking refusal skill-efficacy training among young smokers.

## Data Availability Statement

The raw data supporting the conclusions of this article will be made available by the authors, without undue reservation.

## Ethics Statement

The studies involving human participants were reviewed and approved by Institute of Psychological and Brain Sciences, Zhejiang Normal University. The patients/participants provided their written informed consent to participate in this study.

## Author Contributions

HC: investigation, writing—original draft, and funding acquisition. YF, XL, and LG: writing—review and editing. WL: conceptualization and resources. All authors contributed to the article and approved the submitted version.

## Conflict of Interest

The authors declare that the research was conducted in the absence of any commercial or financial relationships that could be construed as a potential conflict of interest.

## Publisher's Note

All claims expressed in this article are solely those of the authors and do not necessarily represent those of their affiliated organizations, or those of the publisher, the editors and the reviewers. Any product that may be evaluated in this article, or claim that may be made by its manufacturer, is not guaranteed or endorsed by the publisher.
